# The Evolution of Prepubertal Localized Aggressive Periodontitis in Primary and Mixed Dentition—Clinical Evidence

**DOI:** 10.3390/jcm15134874

**Published:** 2026-06-23

**Authors:** Radu-Andrei Moga, Cristian Doru Olteanu, Ada Gabriela Delean

**Affiliations:** 1 Department of Odontology, Endodontics and Oral Pathology, School of Dental Medicine, University of Medicine and Pharmacy Iuliu Hatieganu, Str. Motilor 33, 400001 Cluj-Napoca, Romania; ada.delean@umfcluj.ro; 2 Department of Orthodontics, School of Dental Medicine, University of Medicine and Pharmacy Iuliu Hatieganu, Str. Avram Iancu 31, 400083 Cluj-Napoca, Romania

**Keywords:** juvenile aggressive periodontitis, primary dentition, treatment, mixed dentition, C-MIP, periodontal pocket, bone loss, diagnosis, prepubertal localized aggressive periodontitis

## Abstract

**Background/Objectives**: Prepubertal localized aggressive periodontitis/LPP is an extremely rare but extremely fast-progressing form of periodontal disease involving systemically healthy children, starting in primary and mixed dentition. Our aim is to synthesize the data (January 2014–April 2026) on LPP progression description in systemically healthy children aged 2–13 years; clinical and biological responses to available treatment strategies, focusing on disease progression pattern, treatment efficacy and factors influencing treatment outcomes; and correlating findings with a report of a 24-month follow-up of a female prepubertal Caucasian patient during the primary and early stages of mixed dentition. **Methods**: A total of 489 studies were found after deduplication for the selected period. Due to the eligibility criteria, 9 studies plus another 10 contextual publications were included. Additionally, a 24-month follow-up of a previous LPP case was correlated. **Results**: LPP displayed rapid tissular destruction in primary dentition with risks to transfers to mixed and permanent dentition. The systemic antibiotic treatment reduced tissue loss, enabling fast periodontal regeneration. LPP is rare but severe, with a continuous biological trajectory, and with the window of opportunity remaining when the first symptoms appear. A few months (4–6 months) delay in diagnosis leads to irreversible tooth loss even in young patients with high biological healing potential. **Conclusions**: Systemic antibiotic treatment is mandatory in LPP/C-MIP cases from the primary dentition phase but does not reset host susceptibility. The Amoxicillin/Augmentin–Metronidazole association is recommended, with caution regarding dosage (adverse reactions). Periodontal gains are radiologically and clinically proven, but rebounding is possible.

## 1. Introduction

Prepubertal localized aggressive periodontitis/LPP is one of the rarest forms of inflammatory periodontal disease, still with an incomplete understanding of its etiology and pathogenesis [1,2]. It is mostly known as “localized aggressive juvenile periodontitis, stage III–IV grade C periodontitis in young individuals, and grade C molar-incisor pattern periodontitis/(C-MIP)” [1,3,4,5,6]. It is a chronic inflammatory disease triggered by interactions between microbiome dysbiosis [2,7,8,9,10] and the host immunological response, triggering the loss of tooth-supporting structures (bone and periodontal ligament/PDL), resulting in tooth mobility and loss if untreated [3] in both primary and mixed dentition [11]. It mainly affects the primary first molars and deciduous incisors, with inconsistent biofilm deposits, rapid clinical attachment loss (CALL), and bone destruction [3,11,12,13]. Nevertheless, other teeth have also been reported to be affected by these symptoms (e.g., canine) [14,15].

The cited prevalence seems to be higher in African descendants (7.6%), 1.6% in Israel, 4.3% in the Middle East, 0.3–5.5% in South America [2,3], 2% for African American adolescents and 0.15% for white North American adolescents [16], while for European Caucasian populations, 0.06% [12]–0.1% [17] up to approx. 1% [2,3] has been reported. Moreover, some of the studies seem to report a higher prevalence in females [16,18]. Thus, the reason for the poorly defined epidemiology of LPP is in prepubertal children.

The prepubertal form involves primary (mostly [7]) and mixed dentition, emphasizing the role of early diagnosis and treatments, especially in the context of rapid attachment loss disproportionate to the clinically visible local etiological factors [3,13,14,15,19]. Reports suggest that LPP is an early, clinically detectable manifestation of a lifelong susceptibility phenotype (bone loss in primary dentition signs in 90% of cases diagnosed with localized aggressive periodontitis in the permanent dentition) [7,19].

The reports acknowledged a strong familial aggregation and a bacterial hyperinflammatory profile [7,20], with possible genetic predisposition but without associated systemic conditions [3,14,15]. Clinical symptomatology often goes unnoticed, since in young patients, dental symptoms are often given less attention due to communication difficulties [2,4,14,15,21]. Moreover, there are reports of misdiagnosis (e.g., hypophosphatasia, metabolic disorders, or genetic syndromes) and erroneous treatments at a young age, leading to an early loss of temporary teeth [14,15]. Nevertheless, the same reports suggest bone regeneration and physiological continuation after proper treatment [14,15] in primary dentition. Other consequences can also involve the permanent dentition [3,13,14,15,19]. The pathological mechanism follows a biological continuum spanning across primary dentition (with onset around 2–6), disseminating in the mixed dentition (6–12/13, when the second permanent premolars erupt) to the first permanent molars and continuing in the permanent dentition if untreated. Thus, it is necessary to intercept LPP in the early stages, since that constitutes the clinically critical window for early identification and intervention [14,15,21].

The involved pathological mechanism leads to an extremely rapid progression of bone and periodontal loss (i.e., a “U” shaped bone loss radiological aspect usually appears in a 2 to 4-month interval [12,17,22,23,24]) affecting mostly the primary teeth (i.e., first molars and incisors [5,12,17,25,26,27,28,29,30], canines [14,15,31]), which will be rapidly lost. Various types of investigative methods for bone defects are available, as well as various types of numerical simulations to anticipate potential outcomes of the periodontal defects [32,33]. The onset is usually insidious, especially at very young ages (e.g., 3–4 years old), with little chance of being properly diagnosed and therapeutically addressed [2,14,15]. Usually, the subjective symptomatology involves minor/mild pains and discomforts, and after a short period of time, a variable degree of mobility of the temporary tooth [14,15]. Objectively, no plaque deposits or inflammatory signs are visible. Radiological exams, which are rarely performed due to the young age, display advanced periodontal loss. The tooth is eventually lost in the course of a few months, since the opportunity of interception and treatments in the early stages is often lost [14,15,31].

The difficulties regarding the treatment of LPP reside in the low prevalence of cases [2,3,12] and the young age of the patients, which often leads to misdiagnosis (i.e., various forms of autoimmune disease, metabolic and/or endocrine disorders) [14,15,25,34], frequently leading to the loss of the treatment window of opportunity.

The etiology of LPP is multifactorial and still not completely understood, with reported familial and genetic aggregation [5,8,12,15,26,35,36,37]. There are reports of correlations between prepuberal periodontitis and systemic diseases, residing in general immunodeficiency, with influence over the bodily response to oral bacterial infection [8,14,15]. The prepubertal periodontitis has a general form associated with systemic conditions inducing immunodeficiency (e.g., leukocyte adhesion deficiency, cyclic neutropenia, and other syndromes) and a localized form (LPP) involving systemically healthy children with no identifiable medical disorder [5,14,15,29,38]. The ethiopathological mechanism of LPP resides in a hyper-immune (aggressive autoimmune) response to the periodontopathic bacteria [8,24,25,27,34,38,39,40,41] induced by yet unknown factors (genetic/environmental factors [5,8,14,15,26]) with disruptions in bone metabolism [9]. There are reports of three converging mechanisms that collectively drive disproportionate tissue destruction. It seems that microorganisms such as Actinobacillus actinomycetemcomitans/Aa (particularly its leukotoxic JP2 clone) produce a potent leukotoxin that selectively lyses human leukocytes, disabling the primary innate cellular defense at the periodontal sulcus and enabling unchecked periodontal tissue invasion [5,36,42]. A second mechanism involves hyper-reactive systemic lipopolysaccharides/LPS responsiveness, mediated through amplified Toll-like receptor-4/TLR-4 signaling, which produces disproportionate pro-inflammatory cytokine responses, including elevated interleukin (IL)-6, IL-2, IL-12p70, and MIP-1α, and elevated gingival crevicular fluid/GCF concentrations of the bone resorption mediators RANKL and OPG at both diseased and clinically healthy sites [5,36,42,43,44]. A third mechanism seems to appear during the evolution to mixed dentition by the natural development of new periodontal space around the erupting permanent first molar, which, in the presence of increased bacterial levels and hyperimmune reactivity, will be rapidly colonized, and the risks of new bone loss increase [5,36,42]. The hyper-inflammatory response is triggered by bacteria and their metabolic products (microbial dysbiosis), which will bind to pattern recognition receptors, inducing transcription factor activity and cytokine and chemokine expression [8,9,45]. The clinical laboratory analysis in LLP cases showed increased levels of periodontopathic bacteria (i.e., Actinobacillus actinomycetemcomitans, Fusobacterium nucleatum, Porphyromonas gingivalis, Prevotella intermedia, Treponema denticola, and Tannerella forsythia) and higher levels of B lymphocytes [9,10,15,16,25,45,46,47,48,49].

The difficulty of LPP treatment resides in the rapid progression of the bone loss and the young age of the patients, and often is non-surgical, combining the mechanical debridement with systemic antibiotic therapy [3,5,14,15,17,36,42,50,51,52]. The non-surgical treatment also aims to modulate and modify the host’s hyper-immune response, especially with the adjunctive use of various combinations of Amoxicillin and Metronidazole [3,5,10,14,15,17,29,40,43,44,48,50,53]. The surgical treatment was also reported to be effective with no rebounding in permanent dentition [54]. There are reports of durable clinical parameter improvements across follow-up periods of up to four years when using antibiotic therapy [3,17], as well as when using a surgical approach [54]. The challenges for prepuberal children reside in patients’ cooperation, parental engagement, dose adjustments for body weight, and the absence of a robust randomized controlled trial evidence base specific to this group [3,17]. A recent systematic review report [28] of LPP on primary dentition identified only three interventional studies and twelve case reports, emphasizing the need for more data on this subject.

There is only limited data regarding LPP/prepubertal localized aggressive periodontitis at early ages and its evolution through primary and mixed dentition periods [3,5,14,15,28,40,43,48], which seems to represent sequential stages of a single, chronologically evolving disease process. Moreover, little is known about the optimal dosing, timing, and long-term efficacy of the antibiotic therapy, specifically in the primary dentition [3,14,15,28], due to insufficient data regarding this subject [28,55,56,57,58,59], but also due to the mixed data regarding the effectiveness and outcome of each approach (surgical vs. conservative). New AI technologies are emerging that could increase the LPP bone loss detection.

The present review aims to follow the evolution of prepubertal localized aggressive periodontitis through primary and mixed dentition periods, synthesizing the available published evidence from January 2014 to April 2026 regarding (i) the description of progression of localized aggressive periodontitis in systemically healthy children aged 2–13 years; (ii) clinical and biological responses to available treatment strategies focusing on identifying patterns of disease progression, treatment efficacity and factors influencing the treatments outcome in in terms of bone regeneration, immune modulation, and bacterial load reduction after antibiotic-adjunct non-surgical therapy; and (iii) correlating findings with a report of a 24-month follow-up of a female prepubertal Caucasian patient during the primary and early stages of mixed dentition.

## 2. Materials and Methods

This research aimed to synthesize the evidence on disease progression and treatment response in prepubertal localized aggressive periodontitis/LPP (i.e., Stage III–IV, Grade C, molar-incisor pattern periodontitis) in the primary and mixed dentition (up to 13 years). Additionally, a correlation with an LPP 24-month follow-up case previously reported by our team was performed [14,15]. A narrative design was preferred to a systematic meta-analysis given the heterogeneity in study design, outcome measures, patient populations, available publications, and diagnosis criteria, which precluded meaningful quantitative pooling (with no prospective registration). The quantitative pooling was impossible due to the absence of control groups in all interventional studies, heterogeneity in diagnostic criteria, outcome measurement heterogeneity, sample size inadequacy, population heterogeneity, follow-up heterogeneity, and outcome reporting incompleteness. The research protocol has been approved by the Ethical Committee of the University of Medicine (158/2.04.2018). This review belongs to a larger step-wise research project.

### 2.1. Search Strategy and Databases

A structured electronic literature search was conducted across the following databases: PubMed/MEDLINE, Embase, Web of Science, and the Cochrane Central Register of Controlled Trials/CENTRAL. The publication period (January 2014–April 2026) spanned 12 years. The lower boundary of January 2014 was chosen to capture a full decade of evidence under both the 1999 Armitage classification and the 2018 World Workshop reclassification of periodontitis; the upper boundary of April 2026 corresponds to the date of manuscript preparation, ensuring inclusion of the most recent publications relevant to this topic.

Various search terms were applied in various Boolean combinations across title, abstract, and keyword fields:

- Population and diagnosis terms: “prepubertal periodontitis”, “localized prepubertal periodontitis”, “LPP”, “localized aggressive periodontitis”, “LAP”, “Grade C periodontitis”, “molar-incisor pattern periodontitis”, “C-MIP”, “periodontitis primary teeth”, “periodontitis deciduous dentition”, “periodontitis mixed dentition”, “juvenile periodontitis”, “early-onset periodontitis”.
- Age and dentition qualifiers: “primary dentition”, “deciduous dentition”, “mixed dentition”, “children”, “paediatric”, “prepubertal”, “child periodontitis”, “early onset”.
- Pathogen and biological terms: “Aggregatibacter actinomycetemcomitans”, “JP2 clone”, “bone resorption”, “RANKL”, “OPG”, “lipopolysaccharide”, “GCF cytokines”, “subgingival microbiome”.
- Treatment and outcome terms: “disease progression”, “bone loss”, “attachment loss”, “bone regeneration”, “treatment response”, “treatment outcomes”, “scaling root planning”, “amoxicillin”, “metronidazole”, “non-surgical periodontal therapy”, “antibiotic adjunct”.

The PubMed record before deduplication was 180, while the search Syntax involved the following:

(((“prepubertal periodontitis” [Title/Abstract]) OR

(“localized prepubertal periodontitis” [Title/Abstract]) OR

(“localized aggressive periodontitis” [Title/Abstract]) OR

(“localised aggressive periodontitis” [Title/Abstract]) OR

(“LAP” [Title/Abstract]) OR

(“LPP” [Title/Abstract]) OR

(“molar incisor pattern periodontitis” [Title/Abstract]) OR

(“molar-incisor pattern periodontitis” [Title/Abstract]) OR

(“grade C periodontitis” [Title/Abstract]) OR

(“C-MIP” [Title/Abstract]) OR

(“periodontitis primary teeth” [Title/Abstract]) OR

(“periodontitis deciduous dentition” [Title/Abstract]) OR

(“periodontitis mixed dentition” [Title/Abstract]) OR

(“juvenile periodontitis” [Title/Abstract]) OR

(“early-onset periodontitis” [Title/Abstract]) OR

(“aggressive periodontitis” [MeSH Terms]))

AND

((“primary dentition” [Title/Abstract]) OR

(“deciduous dentition” [Title/Abstract]) OR

(“mixed dentition” [Title/Abstract]) OR

(“deciduous teeth” [MeSH Terms]) OR

(“child” [MeSH Terms]) OR

(“children” [Title/Abstract]) OR

(“paediatric” [Title/Abstract]) OR

(“pediatric” [Title/Abstract]) OR

(“prepubertal” [Title/Abstract]))

AND

((“treatment” [Title/Abstract]) OR

(“therapy” [Title/Abstract]) OR

(“scaling root planing” [Title/Abstract]) OR

(“amoxicillin” [Title/Abstract]) OR

(“metronidazole” [Title/Abstract]) OR

(“antibiotic” [Title/Abstract]) OR

(“disease progression” [Title/Abstract]) OR

(“bone loss” [Title/Abstract]) OR

(“attachment loss” [Title/Abstract]) OR

(“bone regeneration” [Title/Abstract]) OR

(“clinical outcome” [Title/Abstract]))

AND

(“1 January 2014”[Date—Publication]: “30 April 2026”[Date—Publication])

AND

(“English” [Language])).

The search dates were 1 January 2014–30 April 2026 and involved PubMed/MEDLINE, Embase, Web of Science, and CENTRAL. The lower boundary of 1 January 2014 was selected to include both the 1999 Armitage Classification as well as the 2018 World Workshop reclassification. The 30 of April 2026 was the date of manuscript completion.

**Scheme 1:**
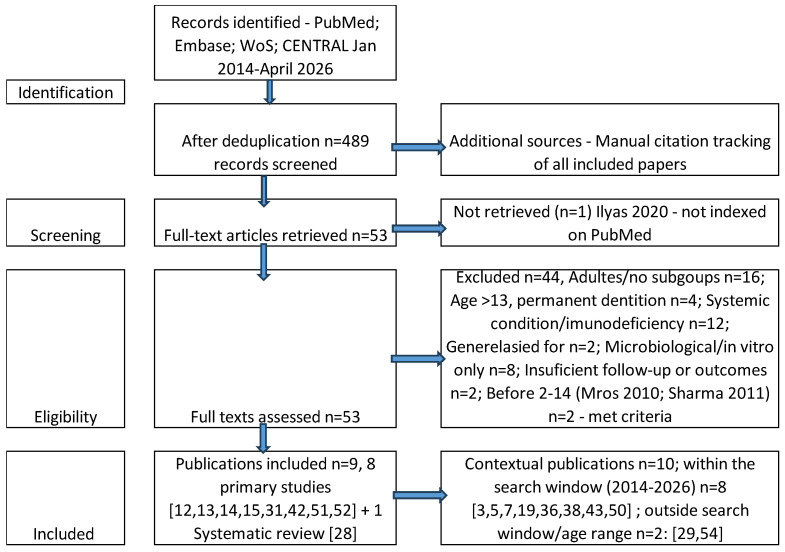
PRISMA flow diagram.

The grey literature was not formally searched, and this is a clear limitation of this review, since relevant unpublished data/conference presentations may exist and were not included herein. The screening was performed independently by two of the authors (clinicians, practitioners, and academics), while disagreements were discussed at a consensus meeting through discussion and reference to the eligibility criteria mentioned below (Scheme 1).

Manual reference list searches of all retrieved full-text articles and relevant systematic reviews were performed to identify additional eligible studies not captured by the electronic search, along with citation tracking.

### 2.2. Eligibility Criteria

Inclusion criteria:

- Population: Systemically healthy children aged 2–13 years (primary dentition ages 2–6; mixed dentition ages 6–13) diagnosed with localized aggressive periodontitis/LPP or its current equivalent (Stage III–IV, Grade C, molar-incisor pattern periodontitis) in the primary or early mixed dentition, without an underlying systemic disease known to predispose to periodontitis (e.g., leukocyte adhesion deficiency, Papillon–Lefèvre syndrome, neutropenia, HIV, hypophosphatasia).
- Study design: Prospective or retrospective cohort studies, non-randomized controlled studies, case series (n ≥ 3), case reports with documented treatment and follow-up, and systematic reviews. No study design was excluded a priori, given the lack of randomized clinical trials/RCT evidence in this population.
- Outcomes reported: At minimum, one of the following was required: (i) quantitative or qualitative data on disease progression (probing depth/PD, clinical attachment level/CAL, radiographic bone loss, tooth exfoliation); or (ii) treatment response data following active periodontal intervention (changes in PD, CAL, bleeding on probing/BoP, plaque index, radiographic outcomes, or biomarker profiles).
- Language: English-language publications only.
- Publication period: January 2014–April 2026.

Exclusion criteria:

- Children with periodontitis as a direct manifestation of a confirmed systemic immunodeficiency or genetic syndrome.
- Studies exclusively reporting on generalized prepubertal periodontitis without a localized subgroup.
- Studies where data for children aged ≤13 years (primary/mixed dentition) could not be separated from data on older adolescents or adults (upper age limit > 13 years with no subgroup-specific data).
- In vitro studies, animal studies, or studies reporting exclusively on microbiological or genetic data without any clinical outcomes.
- Conference abstracts, editorials, and opinion papers without original data.

### 2.3. Study Selection and Data Extraction

Title and abstract screening were performed independently by two of the team members, with disagreements resolved by consensus. Full-text retrieval was performed for all potentially eligible records. Data were extracted into a pre-specified extraction table (Table 1), including the following variables for each included study:

- Author(s), year of publication, journal, and PMID/DOI.
- Study design and, where applicable, clinical trial registration number.
- Country of study conduct.
- Total number of participants and, where reported, the primary/mixed dentition subgroup (age ≤ 13 years).
- Mean age and/or age range.
- Gender distribution (male-to-female ratio).
- Dentition phase at study entry (primary, mixed, or both).
- Treatment strategy or intervention.
- Duration of follow-up.
- Dropouts or loss to follow-up (number and reasons where reported).
- Key outcomes: disease progression (bone loss, attachment loss, tooth exfoliation).
- Key outcomes: treatment response (PD/CAL changes, radiographic bone levels, GCF biomarkers, microbiological outcomes).
- Study limitations and methodological notes.

Given the heterogeneity of the included studies, a narrative synthesis approach was adopted. Quantitative data on clinical parameters (PD, CAL reductions) are presented as reported in the original publications without pooling.

### 2.4. Quality Assessment

Methodological quality of the included studies was appraised using study-design-appropriate tools Table 1, Table 2, Table 3 and Table 4. Prospective and retrospective cohort studies were assessed using the Newcastle–Ottawa Scale (NOS) for cohort studies [55]. The systematic reviews were evaluated using the AMSTAR-2 checklist [56]. Case reports and case series were assessed according to the Joanna Briggs Institute (JBI) critical appraisal checklist for case reports [57]. Quality assessment outcomes were used to contextualize the strength of conclusions drawn from each source; formal quality scoring was not used as an inclusion or exclusion criterion.

The contextual evidence represented a paper that met at least two of the following criteria: (i) reported biological, microbiological, or immunoinflammatory data on the same disease (LPP) in a population with partial overlap with the 2–13 year age interval, providing data directly relevant to interpreting outcomes in the primary included studies; (ii) it was explicitly cited in around two studies of the primary included publications in the systematic review as important evidence for treatment or pathological mechanism; (iii) represented the only available published data on a specific biological domain without which the clinical rationing and understanding is incomplete.

### 2.5. Included Studies

A total of 489 studies were found after deduplication. After the application of eligibility criteria, eight primary publications and one systematic review published between 2014 and 2026 were included in this manuscript (enrolling children within or overlapping the 2–13-year primary/mixed dentition age range). These comprised one prospective non-randomized cohort study reporting a primary dentition subgroup [12], one retrospective descriptive case series characterizing radiographic [51] and clinical features of LPP [13], one systematic review of periodontitis treatment in primary teeth [28], two case reports describing a single 4-year-old Caucasian European patient with delayed diagnosis and subsequent treatment follow-up [14,15], a case report of localized C-MIP in primary dentition of a 5-year-old Indian child with 18-month follow-up [52], a case report of localized C-MIP in primary dentition of a 3-year-old Israelian with 26 month follow-up [31], and one prospective interventional sub-study with a primary/mixed dentition-inclusive cohort evaluating systemic LPS modulation following periodontal therapy [42]. In addition, ten contextual publications from the review period—including a comprehensive narrative review and antibiotic-focused synthesis [3], a microbiome characterization study of C-MIP in primary versus permanent dentition [7], and a systematic review of diagnostic biomarkers in C-MIP [38], a long-term LAP cohort [19], an immunoinflammatory outcomes study [36] and a host-response synthesis [5], a JP2 clone elimination study [43], a microbiome treatment response study [50], a case report of a female teenager with Stage III Grade C periodontitis [29], and a 14–19-year follow-up of 11 children aged 7–13 years treated for LAP, the longest available follow-up dataset for this age group [54]—were also incorporated even though they did not meet the strict primary inclusion criteria due to age range (exceeding 13 years) or study focus (and the scarce evidence found in the current research flow). Some larger cohort studies (age range 5–25 years) had primary or mixed dentition subgroups as discrete strata; thus, only those age-appropriate strata were incorporated into this research.

### 2.6. Follow-Up of a Previous LPP Report (Primary Dentition, Starting from the Age of 4)

**Figure 1:**
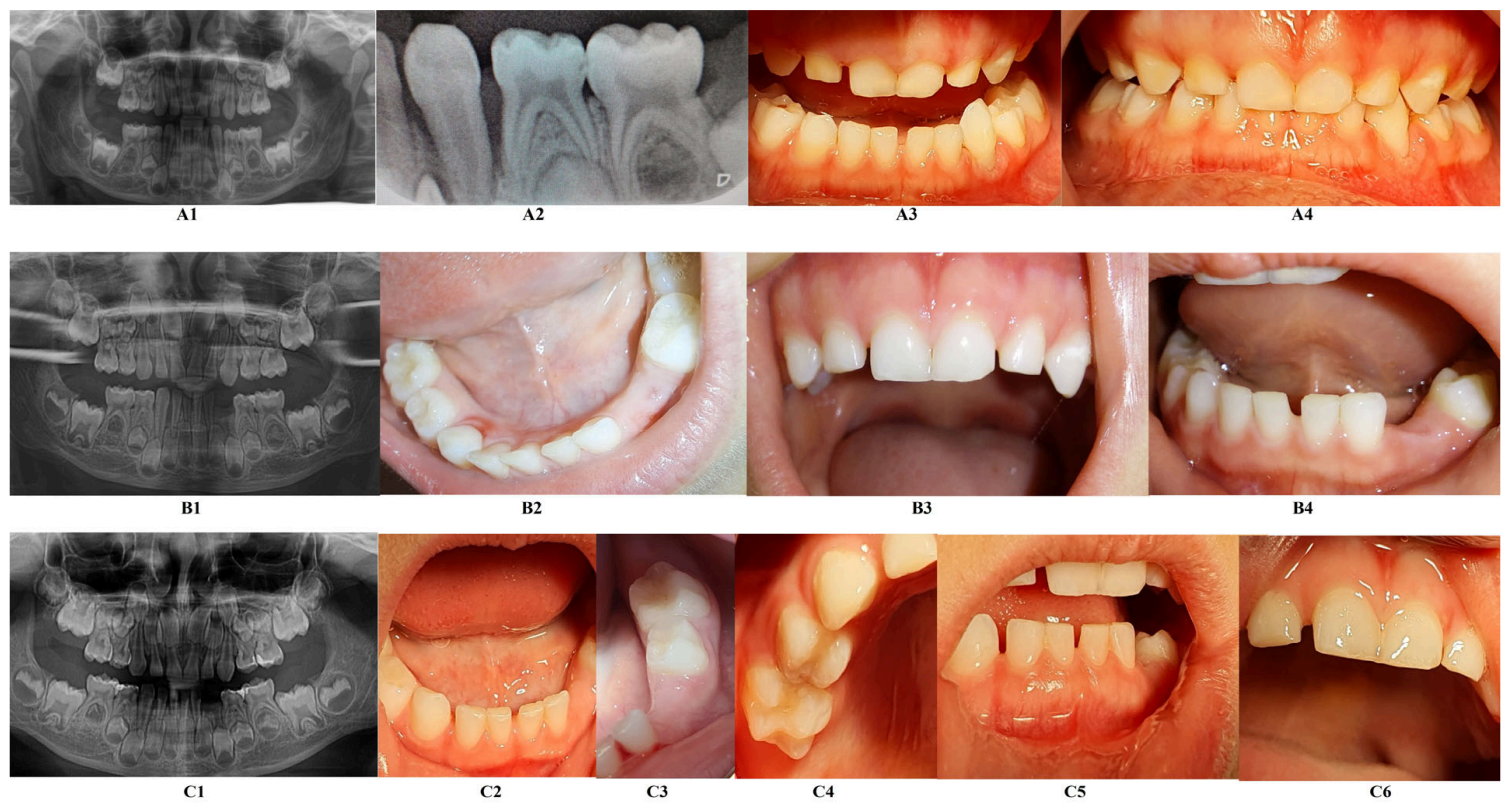
(**A1**–**A4**): June 2023 (**A1**,**A2**)—localized “U” shaped bone loss around 7.3, (**A3**,**A4**)—inflamed periodontium around the lower temporary canine and enhanced movement. (**B1**–**B4**): December 2023 (**B1**)—bone loss around 8.3, upper temporary canine and incisors (i.e., 5.3, 5.2, and 5.1), no radiological signs of definitive canine/3.3 suffering, and with localized visible signs of bone regeneration, (**B2**–**B4**)—clinical examination confirmed the absence of dental plaque, 8.3 advanced mobility, 5.1–5.3 light mobility, and healing of 7.3 area. (**C1**–**C4**): May 2024. C1-panoramic X-ray showed periodontal regeneration and gain of around 8.3 mm, upper and lower incisors. (**C2**–**C6**): clinical examination showed physiological mobility of previously involved teeth, no signs of periodontal inflammation.

Our LPP case involved a 4-year-old (in the spring of 2023) healthy Caucasian girl with an atypical form of prepubertal localized aggressive periodontitis involving the temporary mandibular canines [14,15]. The first subjective and objective symptoms were poor (complaints of mild-to-moderate pain in various parts of the oral cavity) and began in February–March 2023. The case was dismissed by several local dentists, with no clinical objective visible signs. In June 2023, the lower left temporary canine/7.3 began to move, and the case was sent to a university hospital, where, after an X-ray (retro-alveolar and panoramic—Figure 1(A1,A2)), localized “U” shaped bone loss around 7.3 was discovered. The suspicion of metabolic disease (hypophosphatasia/hyperphosphatasia) was first raised, followed by a genetic one. The case was once more dismissed without prescribed therapy, with the recommendation to continue investigating the endocrine path. The blood tests showed only a slight increase in monocytes and lymphocytes and a decrease in neutrophilic granulocytes. In August 2023, due to deteriorating local conditions, another dentist, a university clinician, was consulted (inflamed periodontium around lower temporary canine and enhanced movement, Figure 1(A3,A4)), and based on the patient’s file, the Stage IV grade C localized periodontitis/prepubertal localized aggressive periodontitis/LPP diagnosis was made. Due to advanced clinical signs (i.e., advanced localized periodontal loss and tooth movement), professional hygiene and adjuvant topical applications with Amoxicillin and Metronidazole association around the inflamed periodontal pocket for a period of 10 days were prescribed. The parents were informed about the outcomes and the necessity of treatment. The periodontopathic bacteria test showed high concentrations of Fusobacterium nucleatum/periodonticum and Capnocytophaga. The 7.3 tooth was finally lost in early September 2023. The immunology test showed high levels of IgE (i.e., 828 U/mL when physiological normal is below 60 U/mL) and slight increases for: CPR (i.e., 1.2 mg/dL instead of 1 mg/dL), reticulocytes (i.e., 17 instead of 15 Promille), MPV (11.5 ft instead of 11 ft), eosinophile granulocytes (4% instead of 3%) and monocytes (0.9 G/L instead of 0.77 G/L).

In October 2023, the temporary lower right canine/8.3 showed localized periodontium inflammation and signs of mobility. Another local dentist confirmed the signs and observed slight mobility signs of 5.3, temporary upper right incisors (i.e., 5.2 and 5.1), and temporary lower incisors (i.e., 8.1, 8.2, 71, and 7.2), but no inflamed periodontium. The recommendation was antibiotic treatment with Augmentin of 200 mg (600 mg/day, one spoon three times/day) for 10 days, and one week after the treatment ceased, a periodontopathic bacterial test was taken, which showed negative results.

In December 2023, the family returned abroad to the university clinician who initially diagnosed LPP, and the panoramic X-ray (Figure 1(B1)) confirmed the bone loss around 8.3, upper temporary canine and incisors (i.e., 5.3, 5.2, and 5.1), no radiological signs of definitive canine/3.3 suffering, and with localized visible signs of bone regeneration. Clinical examination confirmed the absence of dental plaque, 8.3 advanced mobility, 5.1–5.3 light mobility, and healing of 7.3 area (Figure 1(B2–B4)). The periodontopathic bacterial test showed highly positive Fusobacterium nucleatum/periodonticum and Capnocytophaga, and mildly positive Treponema denticola, Eubacterium nodatum, and Eikenella corrodens. The professional cleaning was associated with systemic antibiotic therapy (girl’s weight was about 15 kg); Augmentin of 500 mg (1 g/day, 1 tablet twice a day) and Metronidazole of 250 mg (500 mg/day, 1 tablet twice a day, but with the need to adjust the dosage if allergy/overdosed signs were to be displayed) for a period of 10 days. Metronidazole daily dosage was reduced to half (i.e., 250 mg/day divided into two) starting on the second day.

In May 2024, the clinical examination showed physiological mobility of previously involved teeth, no signs of periodontal inflammation (Figure 1(C2–C6)), and the panoramic X-ray (Figure 1(C1)) showed a periodontal regeneration and gain of around 8.3 in upper and lower incisors. The periodontopathic bacteria test revealed the presence of only “precursor germs” of Fusobacterium nucleatum/periodonticum, Campylobacter rectus, Eubacterium nodatum, Eikenella corrodens, and Capnocytophaga spp.

## 3. Results

### 3.1. Literature Search and Study Selection

After deduplication, a total of 489 potentially relevant studies were identified. After title and abstract screening, only 53 full-text articles remained. A further 44 articles were excluded based on the above-mentioned exclusion criteria:

- exclusive focus on adolescents/adults with no primary/mixed dentition subgroups (n = 16);
- patient age > 13 years (permanent dentition; outside the primary/mixed dentition range) with no separation of younger subgroup (n = 4) {specifically: a female teenager aged >13 years in the permanent dentition [29], and a 17-year-old patient [53]};
- periodontitis due to confirmed systemic immunodeficiency, genetic syndrome, or systemic condition (n = 12), including a child with Glanzmann’s thrombasthenia [58] and a case with vitamin D deficiency-associated early-onset periodontitis [59];
- generalized prepubertal periodontitis in primary and mixed dentition (specifically: a case series of two patients with generalized aggressive periodontitis in prepubertal age [41], and an 8-year-old with generalized aggressive periodontitis [37]);
- exclusively microbiological, genetic, or in vitro data (n = 8);
- insufficient follow-up or outcome report (n = 2);
- publications outside the search window (two: a publication with 11 children aged 7–13 years with confirmed LAP and a 14–19-year follow-up, which would have met all population and age criteria [54]; a 4-year-old with premature primary tooth exfoliation and generalized bone loss [21]).

An exhaustive second-round search of reference lists from all included publications, citation tracking, and supplementary PubMed searches using alternative MeSH terms confirmed that no further eligible publications existed within the search window.

Only nine articles (eight primary studies [12,13,14,15,31,42,51,52] and one systematic review [28]) met the initial inclusion criteria; no randomized controlled trials were identified, while the evidence quality was rated overall to be low [28]. Further, ten publications were incorporated as contextual evidence [3,5,7,19,29,36,38,43,50,54].

### 3.2. Characteristics of Included Studies

The nine publications included one systematic review and eight primary studies (Table 5 and Table 6). Two of the studies (a case report and an article [14,15]) described the same 4-year-old Caucasian patient during the primary dentition phase, documenting a delayed-diagnostic phase, progressive dental disease, and temporary tooth loss, followed by the treatment phase, treatment response, and outcome immediately after treatment, but without entering the mixed dentition phase. They are listed separately in Table 1 because they report entirely different clinical data on distinct outcome domains (disease progression vs. treatment response and bone regeneration). Still, they contributed only one patient to the total patient count. Another two studies reported a 3-year-old male [31] and a 5-year-old boy [52].

The eight primary studies and one systematic review enrolled a combined total of 151 patients, where primary or mixed dentition data could be extracted, with ages ranging from approximately 2 to 13 years. Three of the eight primary studies originated from the University of Florida [12,13,42], enrolling exclusively African American children from Tallahassee, Florida, from 2014 to 2018. Two studies are companion papers in the same journal issue, providing complementary radiographic characterization data [13,51]. One study included a patient from Asia (India), adding ethnic and geographic diversity to the evidence base [52]. The 2023 systematic review [28] incorporated data from multiple countries and classification periods.

It should be noted that female patients were predominant in the larger cohort studies, consistent with a predisposition reported in the literature [16,18]. The follow-up ranged between 6 months [42] to 12 months [12,14,15] for the interventional primary studies and up to 5 years for the systematic review [28], while the dropout data were not reported.

### 3.3. Patterns of Disease Progression Across Primary and Mixed Dentition

#### 3.3.1. Radiographic and Clinical Bone Loss in the Primary Dentition

Miller et al. (2018) [13] provided a detailed and systematic characterization of the bone loss patterns for the 33 subjects included in their study. A concentration of the bone loss (mostly vertical) was reported around the primary first molars and incisors [13], mirroring the molar-incisor pattern/C-MIP specific to permanent dentition [6,11]. Various forms of atypical external and internal root resorption were also reported [13] along with furcation involvement and premature exfoliation.

Miller et al. (2017) [19] reported that 90% of young patients with C-MIP permanent dentition displayed signs of bone loss in primary dentition. Additionally, Koo et al. (2024) [7] reported that the dysbiotic microbial signature in primary teeth is microbiologically continuous in permanent dentition.

#### 3.3.2. Disease Transfer to Permanent First Molars During Mixed Dentition

With the physiological age of 6 when the first permanent molar erupts, there seems to be a progressive colonization and infection of the gradually forming gingival sulcus exposed to the oral environment rich in dysbiotic Aa-dominated community from primary dentition. This seems to confirm the three pathophysiological mechanisms [5,36,42,43] previously described.

Koo et al. (2024) [7] reported that in children with active primary dentition C-MIP, the subgingival microbiome of clinically healthy mixed dentition sites shared pathogen community features with diseased sites, suggesting early colonization of the permanent first molar gingival sulcus before clinical signs become apparent. This mechanism has also been reported by Guha Biswas et al. (2023) [28] in their systematic review.

#### 3.3.3. Rate of Progression and Consequences of Untreated Disease

Two articles by Moga et al. [14,15] provided the only prospective real-time documentation of the disease progression in a European Caucasian young child in the context of a diagnostic delay. The two reports confirmed that the primary tooth loss due to LPP/C-MIP can proceed extremely fast (in a few months), thus needing a correct diagnosis and treatment during the window of opportunity period. The initial various subjective symptomatology (common for the four cases in Table 1), ranging from asymptomatic [31] to mild-to-moderate [14,15] and intense pains [52], described by the children before the periodontal tissue destruction, and with no apparent cause, is a sign that should not be ignored. In all these cases, another common factor is the early exfoliation of teeth with advanced periodontal loss. Nevertheless, there is proof that the periodontal gain starts with the reduction of bacterial concentrations, despite the existence of a remaining bacterial reservoir in the oral cavity.

#### 3.3.4. Microbiological Correlates of Disease Progression

Both Velsko et al. (2020) [50] and Amado et al. (2020) [49] reported increased levels of Aa/Aggregatibacter actinomycetemcomitans, Treponema lecithinolyticum, and Tannerella forsythia in subgingival flora with the depletion of health-associated commensals, while Kalash et al. (2015) [42] documented significantly elevated systemic LPS levels at baseline in LAP patients, positively correlated with both the number of diseased sites and GCF RANKL concentrations.

### 3.4. Treatment Strategies Employed

#### 3.4.1. Non-Surgical Mechanical Debridement

All clinical studies [12,14,15,28,42,52] reported only conventional scaling and root planning/SRP treatment (mechanically disrupting the biofilms) associated with systemic antibiotics (Amoxicillin and Metronidazole) due to the Aa presence. No surgical treatment whatsoever was reported for this age group (2–13 years). No Randomized Controlled Trial/RCT was reported. The follow-up period ranged from 3 to 12 months.

#### 3.4.2. Systemic Antibiotic Therapy

The antibiotic combination of Amoxicillin and Metronidazole was reported by all studies [12,14,15,28,31,42,52]. Amoxicillin is recommended for Aa and streptococcal species, while Metronidazole provides selective coverage against strict anaerobes, including P. gingivalis, T. forsythia, and T. denticola. Topical Metronidazole gel, applied during the delayed-diagnosis phase, was reported to be unable to have an impact on disease progression or prevent tooth loss [15]. A recent review report [3] confirmed that systemic delivery is essential for achieving therapeutic concentrations at all oral ecological niches simultaneously. Guha Biswas et al. (2023) [28] also mentioned Tetracycline; however, it should be regarded with care for this age group due to discoloration risks. The recommended dosage is Amoxicillin 500 mg (1.5 g/day) and Metronidazole 250 mg (750 mg/day), three times daily for seven days. For smaller children [14,15], Augmentin (amoxicillin-clavulanate 457 mg/5 mL suspension) or Amoxicillin 750 mg/day [31] associated with Metronidazole suspension for ten days was reported. One of the reports was of a 15-day Amoxicillin–Metronidazole treatment [52]. The Metronidazole dosage was reported to be 250 mg/day [14,15], due to a drug-induced allergy to a 500 mg/day dose, taken on the first day.

#### 3.4.3. Extraction, Space Management, and Supportive Measures

The systematic review [28] reported the extraction of teeth with a hopeless prognosis. Other [14,15] studies reported the involved teeth to be naturally exfoliated. However, if extractions are performed or the exfoliations occur far before the physiological eruption period, space management issues may occur. In all four studies [14,15,31,52], the bacterial reduction seemed to follow the loss of highly mobile teeth, confirming that extraction can help in stabilizing the periodontal tissues.

### 3.5. Clinical Response to Treatment

#### 3.5.1. Probing Depth and Clinical Attachment Level

The primary/mixed dentition group of Merchant et al. (2014) [12] (n = 22, ages 5–12 years) demonstrated statistically significantly greater CAL gains following full-mouth SRP plus Amoxicillin/Metronidazole compared to the permanent dentition group at 3, 6, and 12 months (*p* < 0.05 at all timepoints). The systematic review by Guha Biswas et al. (2023) [28] synthesized outcome data from three non-controlled interventional studies (total n = 60): all reported clinically meaningful improvements in PD and CAL. Studies with the longest follow-up (up to 5 years) demonstrated maintained improvements in compliant patients [28]. The long-term cohort of Miller et al. (2017) [19] reported mean PD reductions of 2.18 ± 1.03 mm and CAL gains of 2.80 ± 1.43 mm sustained across four years. The two studies of Moga et al. (2024) [14,15] reported radiologically visible periodontal gains and physiological mobility for all involved teeth (8.1, 8.2, 71, 7.2, 5.3, 5.2, and 5.1) after the antibiotic treatment.

#### 3.5.2. Impact of Diagnostic Delay and Non-Compliance

The two studies of Moga et al. (2024) [14,15] reported that a 4 to 6-month delay in diagnosis and treatment resulted in missing the window of treatment opportunity and irreversible periodontal loss that led to the temporary canine loss. A similar aspect was provided by the other reports [12,42,51] with inferior outcomes for non-compliance with the antibiotic treatments. Mass et al. (2018) [31] displayed similarities with Moga et al. (2024) [14,15] when it came to the response to the treatment.

### 3.6. Post-Treatment Outcomes in Primary and Mixed Dentition: Bone Regeneration, Immunoinflammatory Modulation, and Bacterial Load Reduction

#### 3.6.1. Radiographic Bone Regeneration

It must be acknowledged that the bacterial suppression removes the primary inflammatory stimulus, which reduces the RANKL-driven osteoclastic signal and creates the biological conditions for periodontal regeneration. Moga et al. (2024) [14,15] reported periapical bone regeneration around primary teeth (a Caucasian girl patient aged 4) after two months of systemic antibiotic treatment. The regeneration speed (radiologically detectable after around 8 weeks) reflects the unique osteogenic biology of immature alveolar bone: higher endogenous concentrations of BMP-2, BMP-7, IGF-1, and TGF-β, combined with higher cellular turnover and greater vascularity, facilitate rapid mineralization once osteoclastic bone resorption is arrested [5,36,42,43,44]. The radiological stability was confirmed at a 12-month interval [14,15]. Miguel et al. (2025) [3] reported the lack of systematic quantitative radiographic bone level measurement for this age group.

#### 3.6.2. Immunoinflammatory Response to Antibiotic-Adjunct Therapy

Branco-de-Almeida et al. (2021) [36] evaluated GCF biomarker profiles at baseline and at 3, 6, 12, and 24 months in 66 patients (ages 5–25 years) following SRP associated with Amoxicillin/Metronidazole. GCF levels of IL-12p70, IL-2, IL-6, MIP-1α, RANKL, and OPG were all significantly reduced at diseased sites after the treatment. The discriminant GCF cytokine profile that differentiated diseased from healthy sites at baseline was progressively attenuated over 24 months [36]. The RANKL/OPG ratio (i.e., elevated in GCF of diseased sites at baseline) was significantly reduced at multiple timepoints, directly reflecting suppression of the osteoclastic drive responsible for bone destruction [36]. Kalash et al. (2015) [42] demonstrated significant reductions in systemic serum LPS at 6 months after the treatment, correlated with PD and CAL improvements. Allin et al. (2016) [44] demonstrated that the baseline LPS hyper-responsive phenotype predicts the magnitude of treatment response but partially rebounds at 6–12 months, indicating that the constitutively amplified TLR-4 signaling capacity is not permanently corrected by a single treatment cycle. In the mixed dentition context, this rebound means that as new permanent teeth erupt into a susceptible host environment, continuous periodontal surveillance is required throughout the mixed dentition phase.

#### 3.6.3. Bacterial Load Reduction and Subgingival Community Restructuring

Velsko et al. (2020) [50] demonstrated that following full-mouth SRP plus systemic Amoxicillin/Metronidazole, diseased-site communities shifted progressively towards the health-associated profile at 3–24 months post-treatment, with Aa. showing the largest proportional reduction. Burgess et al. (2017) [43] demonstrated near-complete elimination of the JP2 clone from both diseased sites (*p* < 0.0001) and clinically healthy sites (*p* < 0.0001), confirming that the combined protocol suppresses the full oral reservoir of this pathogen. This full-mouth JP2 reservoir suppression is of particular strategic importance in mixed dentition patients: permanent first molars erupting into an oral environment with a substantially reduced total JP2 burden are less likely to undergo rapid periodontal colonization [43]. Amado et al. (2020) [49] demonstrated that Aa abundance is 50 times higher at deep periodontal sites than in controls, extending to clinically healthy and supragingival sites, reinforcing the mechanistic necessity of systemic antibiotic delivery to suppress (i.e., as best as possible) all oral and systemic reservoirs simultaneously.

Thus, based on above-mentioned (i.e., microbiological, immunoinflammatory, and radiographic) evidence, a visible pattern can be displayed for the primary and mixed dentition with LPP evidence: systemic antibiotic treatment induces fast suppression of Aa (including JP2 clone) in the oral cavity within 3 months; followed by a progressive community restructuring towards a health-associated profile by 6–12 months; with a reduction in GCF pro-inflammatory cytokines, RANKL/OPG ratio, and systemic LPS, and creating a reduced inflammation environment; along with disappearance/reduction in osteoclastic bone resorption, and enabling osteoblastic mineralization; and with radiographically detectable bone regeneration and clinical stability within 12–24 months for compliant patients.

### 3.7. Follow-Up of the European Caucasian Patient for a Period of Another 24 Months (May 2024–April 2026)

Our follow-up herein continues the above-mentioned history presented in the previous two articles [14,15].

**Figure 2:**
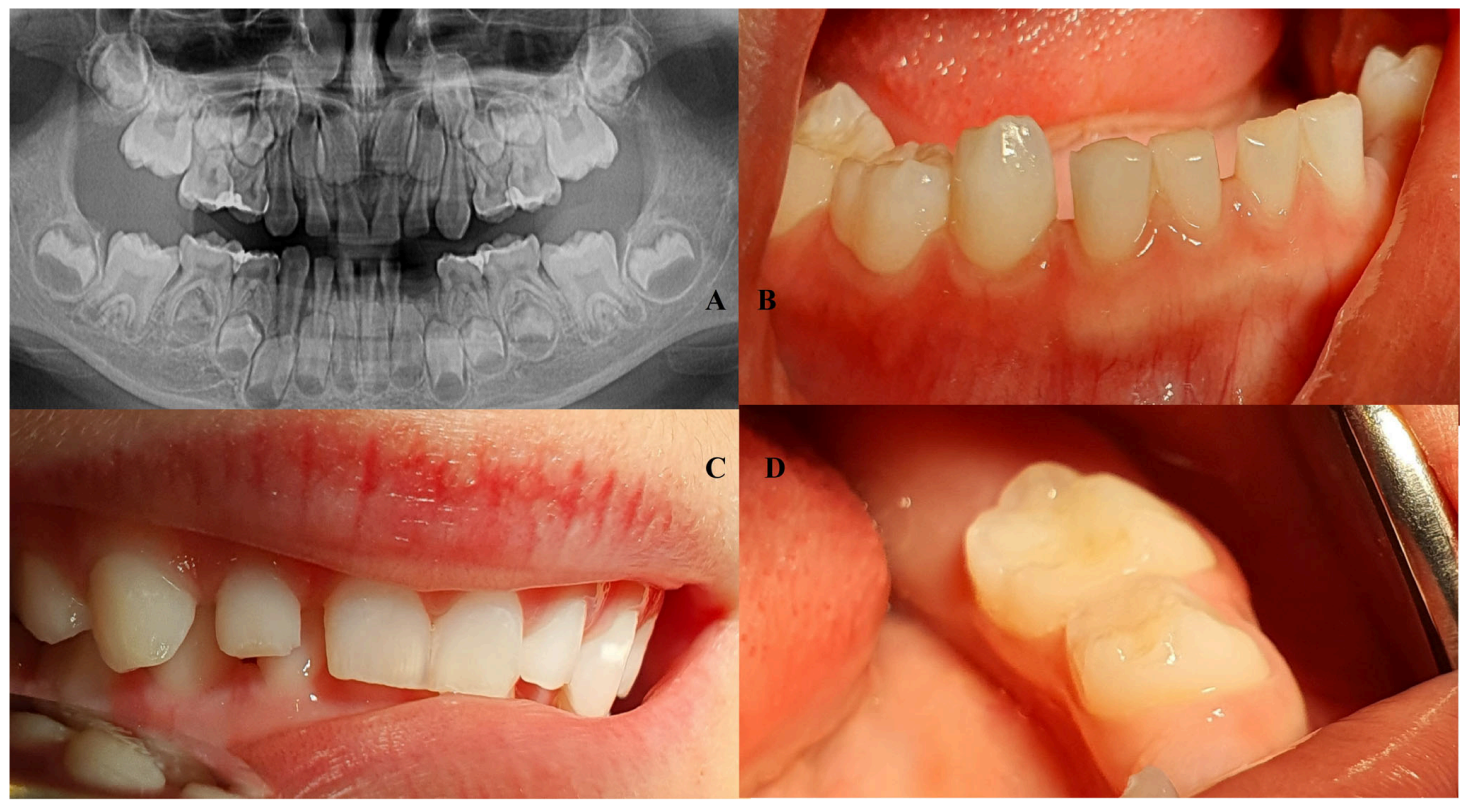
December 2024: (**A**)—panoramic X-ray showed visible continuous periodontal regeneration around all involved areas, (**B**–**D**)—clinical examination revealed that physiological mobility of previously involved teeth was guarded with no signs of periodontal inflammation.

In December 2024, the family consulted the university clinician abroad for a 6-month follow-up. The physiological mobility of previously involved teeth was guarded with no signs of periodontal inflammation (Figure 2B–D), while the panoramic X-ray (Figure 2A) showed visible continuous periodontal regeneration around all involved areas (visible when compared with the previous one, taken May 2024/Figure 1(C1)).

**Figure 3:**
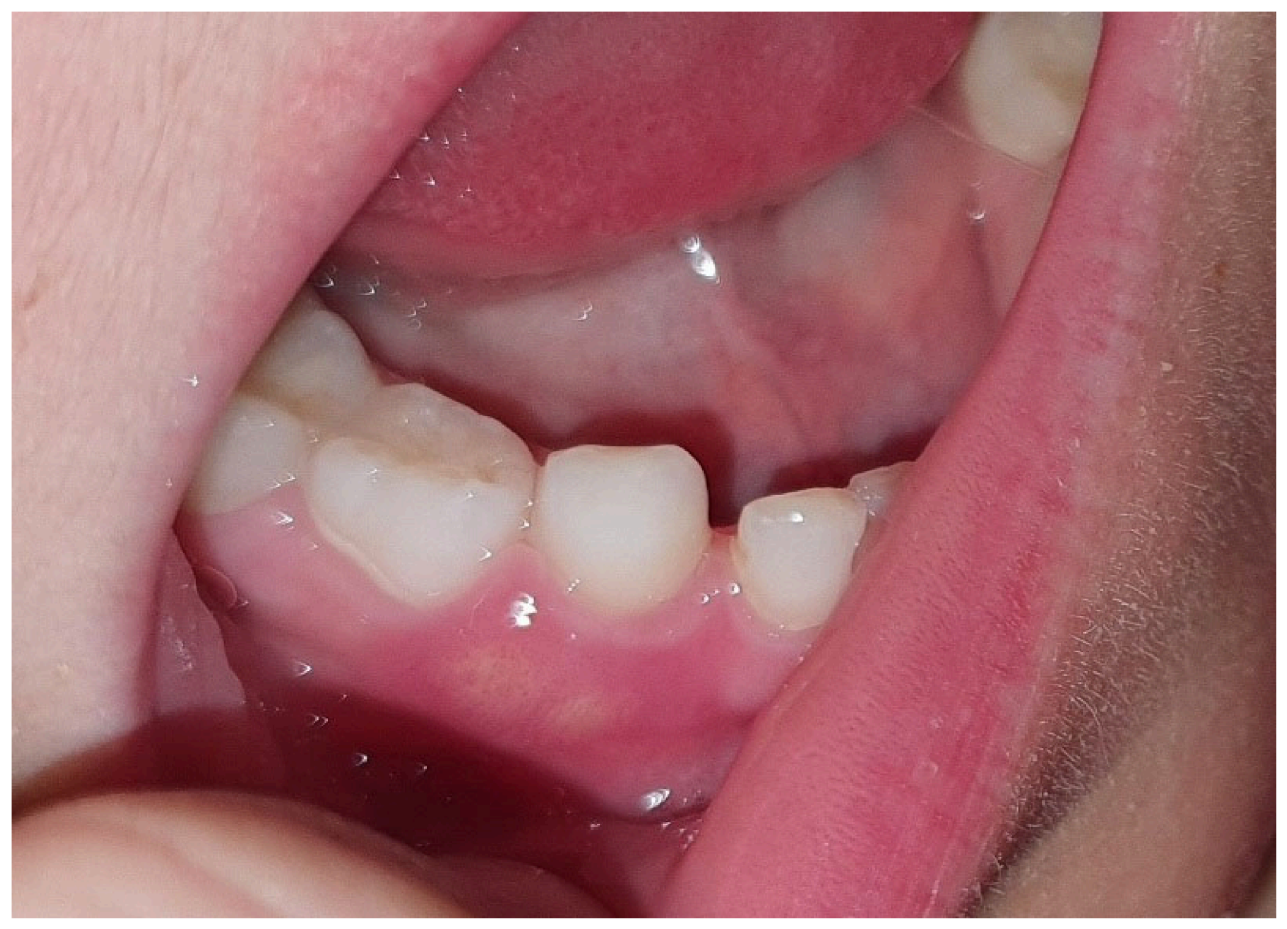
Early February 2025—a vestibular periodontal abscess located around 8.3 developed.

In early February 2025 (being in her hometown), a vestibular periodontal abscess located around 8.3 developed (Figure 3). No treatment whatsoever was performed. In late March 2025, after the resolution of the acute process, another periodontopathic bacteria test (i.e., PET plus), performed by a hometown local dentist, revealed the presence of Aggregatibacter actinomycetemcomitans/Aa in a higher quantity and Capnocytophaga gingivalis in a smaller quantity. No other germs were detected (despite the other ones found in late May 2024—Fusobacterium nucleatum/periodonticum, *Campylobacter rectus*, *Eubacterium nodatum*, *Eikenella corrodens*, and *Capnocytophaga* spp). The recommended antibiotic treatment was Amoxicillin 1.5 g/day (3 × 500 mg/d), for 7 days. However, the parents insisted, and with the recommendation of the university clinician abroad, the Metronidazole (250 mg/day divided into two) for 7 days was added.

**Figure 4:**
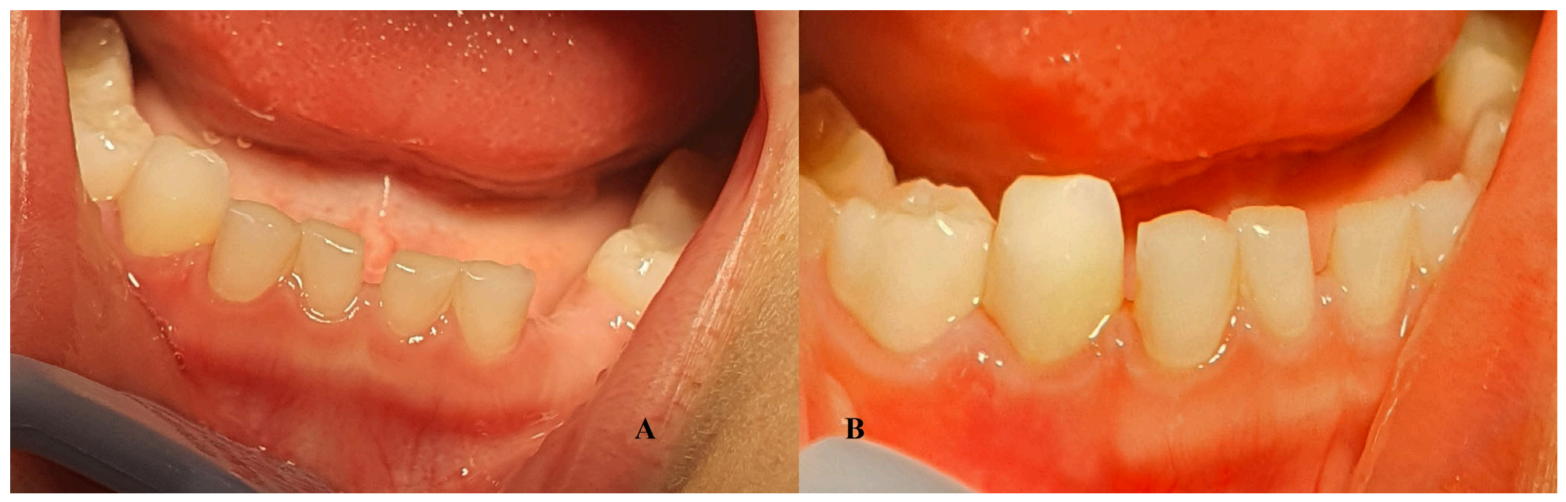
Late April 2025: (**A**,**B**)—visible periodontal loss around 8.3, but no other signs of periodontal inflammation, and the first permanent molars eruption was noticed (the beginning of the mixed phase dentition).

In late April 2025, the family visited the university clinician abroad, and the clinical examination showed a visible periodontal loss around 8.3, but no other signs of periodontal inflammation (Figure 4A,B), and the first permanent molars eruption was noticed (the beginning of the mixed phase dentition).

**Figure 5:**
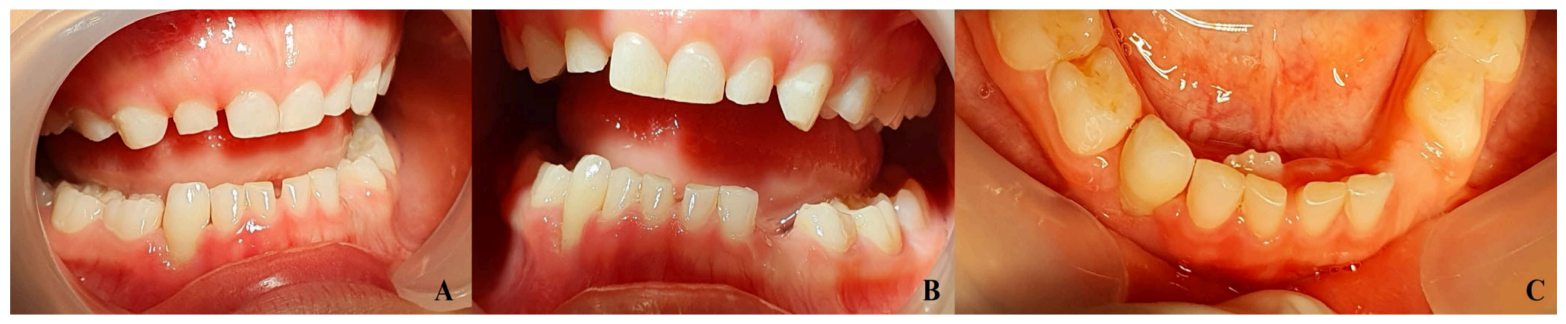
Late August 2025: (**A**–**C)**—confirms the periodontal loss around 8.3, the lack of signs of periodontal inflammation, and the beginning of 4.1 eruption.

In Late August 2025, a new clinical examination (Figure 5A–C) confirmed the periodontal loss around 8.3, the lack of signs of periodontal inflammation, and the beginning of 4.1 eruption, Figure 5A–C. In late September 2025, the 8.3 was finally lost.

**Figure 6:**
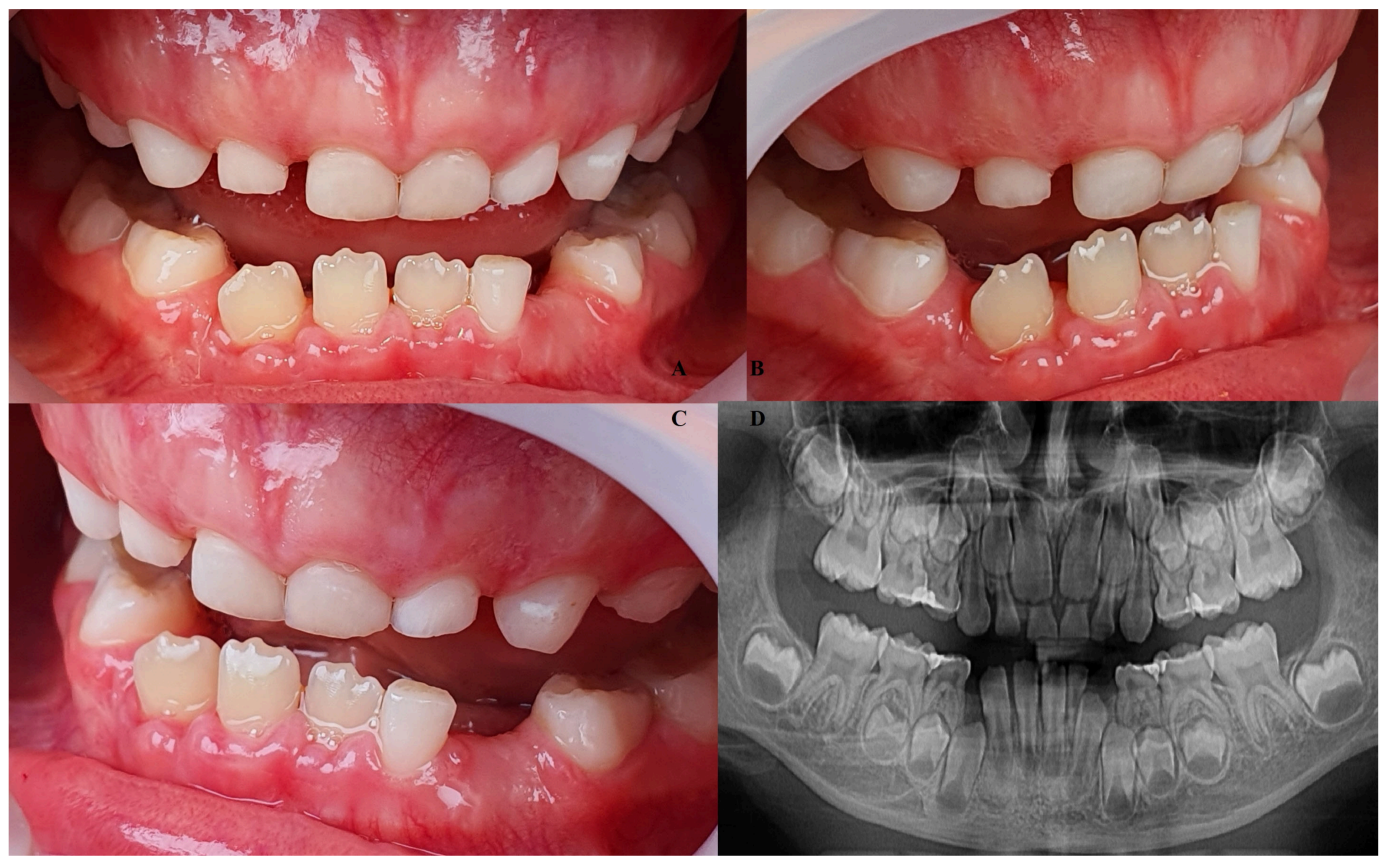
Early January 2026 (the patient now aged 7): (**A**–**C**)—the presence of 4.1, 4.2, 3.1, and the imminent physiological loss of 5.1 and 6.1; (**D**)—X-ray confirmed the lack of periodontal loss around the first definitive molars and incisors and periodontal gain after the 8.3 loss.

In early January 2026, the patient, now aged 7, attended another follow-up with clinical and radiological control (Figure 6A–D). The radiological X-ray confirmed the lack of periodontal loss around the first definitive molars and incisors and periodontal gain after the 8.3 loss (Figure 6D). The clinical examination showed the presence of 4.1, 4.2, 3.1, and the imminent physiological loss of 5.1 and 6.1 (Figure 6A–C).

**Figure 7:**
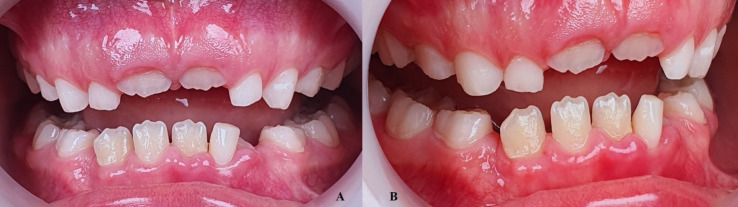
Mixed dentition phase, early April 2026: (**A**,**B**)—eruption of 11, 21, and 3.2, and imminent physiological loss of 7.2.

In late April 2026, the next follow-up noticed the eruption of 11, 21, 3.2, and imminent physiological loss of 7.2 (Figure 7A,B). No periodontal inflammation signs were noticed.

**Figure 8:**
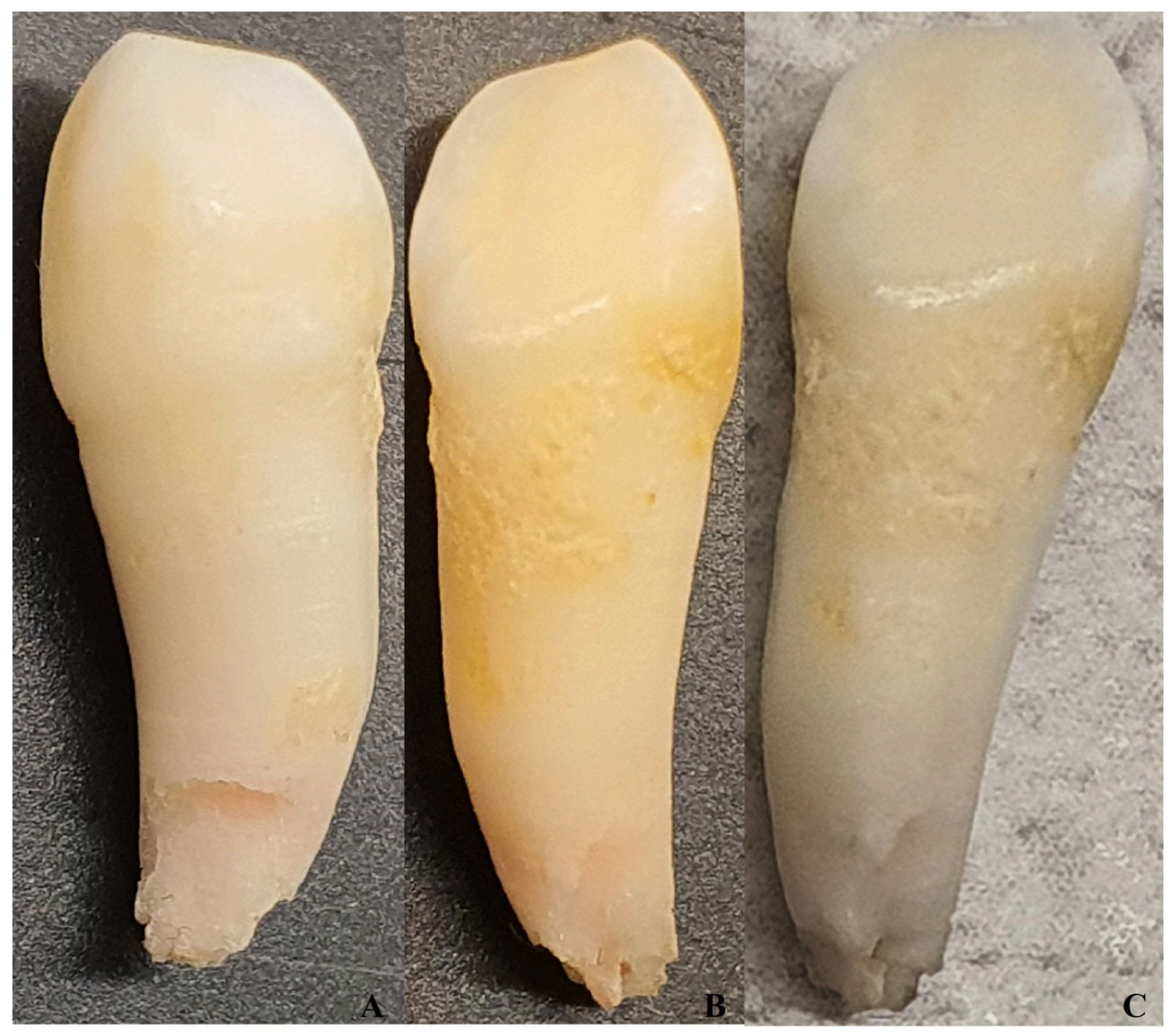
The 7.3 primary/temporary canine, lost in autumn 2024, with visible signs of apical third external root resorption: (**A**)—vestibular view, (**B**,**C**)—lingual proximal view.

Another aspect to note is that both 7.3 (Figure 8A–C) and 8.3 lost due to LPP (early exfoliation), displayed apical third root resorption.

## 4. Discussion

This study investigated the evidence found in current research from January 2014 to April 2026 on prepuberal localized aggressive periodontitis/LPP/C-MIP ([1,2,3,4,5,6]) in systemically healthy children aged 2–13 years [3,5,7,12,13,14,15,19,28,36,37,38,42,43,50,51,52] using specific standards [55,56,57]. Additionally, the data collected from a 24-month follow-up of a Caucasian female patient previously investigated [14,15] were integrated.

### 4.1. Current Research Flow Literature Correlations

The evidence showed that if untreated, a fast localized bone and periodontal loss appears and progresses within weeks, leading to tooth loss [12,13,14,15,28,31,42,51,52]. Both the primary and mixed dentition phases allow systemic antibiotic treatment, while the early diagnosis represents a critical window of opportunity for controlling and stopping the tissue loss [12,17,20,22,23,24], as well as reducing the risks of progression to permanent first molars [12,13,14,15,28,42,51,52]. The treatment recommended for this age group is the common SRP associated with systemic antibiotic treatment (associations of Amoxicillin/Augmentin and Metronidazole), with better CAL gains when compared with other groups due to osteogenic and immunological advantages of immature alveolar tissue [3,5,7,19,31,36,38,43,50]. Nevertheless, there are some issues related to the lack of RCT; most of the data are related to the African American population (where the prevalence is at the highest level, about 2% [2,3,12,16,17,18]), while the quantitative radiographic bone measurements were not available [12,13,14,15,28,42,51,52].

LPP/C-MIP seems to display a unified continuum pattern starting in the primary dentition, continuing in the mixed dentition phase, and continuing its evolution during the permanent dentition phase [12,13,14,15,28,41,42,51,52]. There are reports of 90% of permanent dentition C-MIP patients displaying bone loss through the primary dentition phase [19]. Other studies reported both the presence of a continuous dysbiotic bacterial community in the oral cavity, starting from the primary dentition phase [7], and a host susceptibility phenotype (i.e., TLR-4 LPS responsiveness) [39,44]. Based on the above-mentioned, the mixed dentition phase seems to be the transitional period of disease and bacterial propagation from the primary teeth to the permanent teeth (especially permanent first molars), creating proper conditions for new attachment loss if systemic antibiotic treatment during early phases is neglected [12,13,14,15,28,31,42,51,52].

Another issue that must be seriously taken into consideration for the LPP/C-MIP long-run treatment is the 6–12 month rebound after systemic antibiotic treatment [44], also in line with our herein case report [14,15] of previous evolution as well as for the 24-month follow-up period. Nevertheless, another report [31] for a 24-month follow-up of a 3-year-old male reported no rebound and only an early exfoliation of the primary canines. Moreover, it seems that an older study of Mros et al. (2010) [54] disagrees with the above-mentioned, reporting that even in the absence of formal periodontal maintenance, the recurrence in permanent dentition does not occur. However, its protocol [54] included the extraction prevailing over the conservative SPR-systemic antibiotic treatment, eliminating the infected sites as soon as possible in order to limit the spread of tissue loss and bacterial colonization. This study [54] also reinforces the idea of early diagnosis and interception of the LPP/C-MIP as extremely important for the outcome.

Thus, it seems that systemic antibiotic treatment does not reset host susceptibility (also in line with [5,8,9,30,36,42,43,44,45]), requiring active surveillance (i.e., clinical, radiological, and bacterial tests) and long-term treatment for the prepubertal localized aggressive periodontitis patients. The end of the mixed dentition phase (12–13 years) represents a formal clinical checkpoint for periodontal reassessment in cases with a history of LPP/C-MIP.

Reports in Table 5 and Table 6 suggest that the SPR treatment could and/or should be associated with systemic antibiotic treatment (Amoxicillin/Augmentin–Metronidazole) from the primary dentition phase, following the rationale of the three ethiopathological converging mechanisms of LPP that collectively drive the disproportionate tissue destruction [5,8,9,12,14,15,24,25,26,27,29,34,35,36,38,39,40,42]. Our 24-month follow-up case is consistent with this observation, as well as in line with Mass et al. (2018) [31] report. The dosage reported in the Table 5 studies [12,13,14,15,28,42,51,52] as well as by Miguel et al. (2025) [3] seems to be up to Amoxicillin 1.5 g/day (3 × 500 mg/d) and Metronidazole 750 mg/day (3 × 250 mg/d) for 7 days. Mass et al. (2018) [31] cited a lower dosage of Amoxicillin of 750 mg/day (3 × 250 mg/d) for the same period of 7 days, for a 3-year-old. Amoxicillin can also be replaced by Augmentin, following the same dosage [14,15]. However, regarding the Metronidazole dosage for prepubertal patients, due to known adverse reactions (also reported in [14,15]), the 750 mg/d could in fact be much lower (250 mg/d) and still be effective as previously reported [14,15]. The time range for the systemic antibiotic treatment is 7 [31]–10 [14,15]–15 [52] days.

Early interception and diagnosis of LPP/C-MIP are mandatory for being able to use the treatment windows of opportunity, since the rapid periodontal tissue loss occurs in a few months [14,15]. For the diagnosis, radiological exams associated with early interception of the clinical objective and subjective (also of extreme importance) symptoms and bacterial tests are mandatory, since lack of family awareness and natural difficulties in prepubertal age cooperation. Since the fast progression of the tissular loss, the antibiotic systemic treatment protocol could be initiated without awaiting the genetic or metabolic investigations [14,15]. The blood samples can also help in confirming the clinical diagnosis, as reported in the clinical case herein [14,15,31,52]. Moreover, the C-MIP pattern is not always followed, and other teeth can be involved in LPP (e.g., temporary canines [14,15,31]).

Another issue that must be addressed is related to the ethnic, geographic, and socioeconomic considerations, since a higher prevalence is reported for African American children (around 2%), while for white European Caucasians around 0.15% [2,3,12,16,17,18]. Moreover, most of the data reported in the current research flow is focused on populations with the highest prevalence (as displayed by Table 1). Thus, for European Caucasians, the evidence is scarce; only three reports were found, covering the prepubertal age with the primary teeth phase [14,15] and adolescence with permanent dentition [29], while herein, the 24-month follow-up case covers the mixed dentition period. Together, these three above-mentioned constitute clinical evidence for European Caucasian LPP cases, confirming that clinical course and response, combined with the systemic antibiotic treatment, follow the same pathway as for the African American populations.

The limitations of this study are related to the fact that no RCT data were found for this primary and mixed dentition age group. Moreover, there is a small sample size (the largest primary/mixed dentition-specific sample of 33 [13], and interventional data of 22 for primary dentition [12]), consistent with the reported low prevalence, lack of radiological measurements of the bone level, focus of most of the data on the African American populations, and short follow-up of 6–12 months.

### 4.2. Follow-Up Case Report Correlations

In both temporary canines lost to LPP (early exfoliation), some apical-third root resorption was present (Figure 8), which is consistent with two reports [6,11]. Despite the location of both temporary canines being unusual when compared with the most common C-MIP pattern, the vertical bone destruction (“U” shape) is consistent with the Miller et al. (2018) [13] report.

The evolution of this atypical case of LPP, during the 24-month follow-up, seems to follow the clinically reported pattern [13,19,31] as well as the physiopathological mechanisms in other studies [5,36,42,43]. The periodontopathic bacteria tests performed during this period of two years also seem to confirm the findings of Koo et al. (2024) [7] and Guha Biswas et al. (2023) [28] regarding the dysbiotic microbial signature in primary teeth, as well as the presumed colonization of the gradually formed new gingival sulcus around the permanent dentition. The specific bacterial types present in the oral cavity seem to be in line with those reported by Velsko et al. (2020) [50] and Amado et al. (2020) [49].

It must be emphasized that since the host hyper-immune mechanism cannot be changed (especially at such a young age), the only treatment possibility to contain the auto-destructive body response resides in managing the trigger factor (i.e., the quantity and types of periodontopathic bacteria present in the oral cavity) [15]. Thus, the medical reasoning applies for multiple bacterial tests.

The treatment included SPR and systemic antibiotic associations of Amoxicillin/Augmentin and Metronidazole, in line with Table 5 [12,14,15,28,31,42,52] and other reports [3,5,10,17,29,36,40,43,48,50,51,53]. The dosage was different, however, with Amoxicillin 1.5 g/day (3 × 500 mg/d), for 7 days and Metronidazole (250 mg/day divided into two) for 7 days. Table 5 reports the 1.5 g/day of Amoxicillin (as in our case), but 750 mg/day of Metronidazole [12,42] vs. 250 mg/day of Metronidazole in our case. The difference is that after an initial 500 mg/day of Metronidazole (below the dosage recommended by the manufacturer for this specific age group) was prescribed and taken on the first day, the patient displayed a drug-induced allergy [14], which forced us to reduce the dosage in half. Thus, to avoid any other complications related to this drug, we decided to prescribe the already proven safe dosage.

The extraction of the involved teeth (7.3 and 8.3) was not an option due to the young age of the patient and the parents’ reluctance, despite other studies’ reports [28]. Herein, the conservative approach was, however, confirmed by other reports [31,52] (Table 1).

The importance of antibiotic treatment and its benefits in radiologically confirmed fast periodontal regeneration are confirmed by the evolution of the LPP case in our study. Nevertheless, the rebound found during the follow-up interval is consistent with the observations reported by Allin et al. (2016) [44] regarding the 6–12 months rebound interval, as well as the need for permanent assessment of the case for preventing permanent dentition damage. In this strategy, the bacterial tests are mandatory [9,10,15,16,21,25,45,46,47,48,49].

Nevertheless, some recommendations can be drawn from the above-mentioned evidence: when unexplained primary tooth mobility and/or pains occur associated with or without gingival inflammation disproportionate to visible biofilm, the patient should immediately undergo radiological examination, full-mouth probing, and basic blood sample tests. As soon as these previous signs are positive and the radiological examinations confirm the suspicion of LPP, a full-mouth SPR associated with systemic Amoxicillin/Augmentin and Metronidazole treatment for 7–10 days could or should be initiated immediately. Before this, a bacterial periodontopathic sample should be collected to identify the main bacterial types involved. Regarding the antibiotic systemic treatment, the dosage must be taken into consideration, as well as the pediatric recommended dose adjustment, with particular care regarding the Metronidazole dosage. After the initial systemic antibiotic treatment, following the next month (3, 6, and 12 months), clinical and radiological reassessment should be performed. Due to the above-mentioned rebound evidence, continuous annual monitoring through the mixed dentition phase should be performed, with particular attention to the first permanent molar eruption (around the age of 6) up to the second premolar eruption (around the age of 11–13), with a complete reassessment when entering the permanent dentition phase. When dealing with teeth with complete loss of periodontal support, the extraction should be taken into consideration (due to displayed clinical evidence of periodontal regeneration afterwards), if the conservative approach fails.

An aspect that should not be minimized is communication between parents/family, especially if dealing with previous negative medical experiences, where varying degrees of mistrust seem to be natural. Every time, a rational, scientific, evidence-based approach is advised.

This study also emphasizes the need for a multi-center double-blind RCT comparing the full-mouth SRP associated with systemic antibiotic treatment in LPP/C-MIP cases with primary and mixed dentition, with radiological measurements and more than 3 months of follow-up. Additionally, the dosage of the systemic antibiotic treatment should be validated by a well-designed protocol. Furthermore, the periodontal defects can be radiologically assessed, and the numerical analyses can be used to create various developments in treatment scenarios [32,33]. The newly introduced AI models could increase the early detection of bone loss in LPP cases; thus, studies in this direction could be created.

## 5. Conclusions

1. LPP in primary and mixed dentition is rare, especially in European Caucasian children, but severe, starting with the eruption of primary dentition, with what seems to be a continuous biological trajectory, and with the window of opportunity remaining when the first symptoms appear.
2. A diagnosis delay of a few months (4–6 months) seems to lead to irreversible tooth loss even in young patients with a high biological healing potential, suggesting that timely diagnosis is the single most modifiable determinant of the clinical outcome.
3. It seems that systemic antibiotic treatment during the primary dentition phase of LPP does not reset host susceptibility, requiring active surveillance (i.e., clinical, radiological, and bacterial tests) and long-term treatment for the pre-pubertal localized aggressive periodontitis patients.
4. It seems that systemic antibiotic treatment (Amoxicillin/Augmentin–Metronidazole) is of great importance in LPP cases from the primary dentition phase, as also reported by our 24-month follow-up.
5. The current literature reported dosage for the systemic antibiotic treatment is up to 1.5 g/d Amoxicillin/Augmentin and 250–750 mg/d Metronidazole, for 7–10 days. Nevertheless, the Metronidazole dosage must be under careful surveillance due to known adverse reactions, especially for prepubertal patients.
6. The periodontal gains after the systemic antibiotic treatment seem to occur rapidly, reflecting the osteogenic advantages of immature alveolar bone and the pre-pubertal microbiological environment. The periodontal gains proved that even at a young age, under adequate treatment, LPP can be kept under control, providing correct and physiological development.
7. Nevertheless, the data provided by the current research flow is extremely low, emphasizing the need for high-quality clinical trial evidence, as well as individualization of treatment for every LPP case.

## 6. Clinical and Scientific Significance

The prevalence of the localized prepubertal aggressive periodontitis/LPP/C-MIP is extremely low worldwide (highest of 2% being in African American populations), with a maximum reported rate of 0.15% in European Caucasians. There is little data on LPP in primary and mixed dentition (most of it belonging to the African American population); thus, this study fills the gap in the scientific literature for European Caucasian children. Our study rationalizes and synthesizes the available clinical knowledge (January 2014–April 2026) of the LPP pathological mechanism, describes its evolution in primary and mixed dentition, and outlines the required treatment and its outcome. The type of systemic antibiotic treatment based on the bacteriological test and the daily dosage is also presented, which is of extreme importance for daily clinical treatment. It also displayed the consequences of LPP misdiagnosis and lack of treatment with the fast progression of the bone and periodontal loss in an interval of around 4–6 months. These were correlated with a 24-month follow-up of a pre-pubertal patient through the primary and mixed dentition phase, describing the antibiotic dosage, the types of bacteria found, the rebound, the coverage of the rebound phase, and the radiological proof of systemic antibiotic treatment efficacy, displaying the periodontal gains. By correlating the findings in our follow-up case with current reports, we were able to perform some clinical observations related to the type of antibiotic (Augmentin vs. Amoxicillin) and the dosage of Metronidazole (250 mg/d vs. 750 mg/d), as well as for the period (7 vs. 10 days).

## Figures and Tables

**Table 1:** Newcastle–Ottawa Scale/NOS cohort studies—both studies have significant risks of bias due to the absence of a control group (the most important limitation) and incomplete dropout reporting. Kalash’s 6-month follow-up is insufficient to assess the sustained treatment effect.

Domain	Item	Merchant 2014 [12]	Kalash 2015 [42]
**Selection**	Representativeness of exposed cohort	yes (selected community sample)	yes
	Selection of non-exposed cohort	no (no unexposed/control group)	no
	Ascertainment of exposure	yes (standardized clinical protocol)	yes
	Outcome not present at start	yes	yes
**Comparability**	Comparability on design/analysis	no (no control group; no confounder adjustment)	no
**Outcome**	Assessment of outcome	yes (calibrated examiners)	yes
	Follow-up length adequate	yes (12 months)	no (6 months; short)
	Adequacy of follow-up	no (dropout not fully reported)	no
**Total**		**5/9—Moderate**	**4/9—Low-Moderate**

**Table 2:** JBI Critical appraisal checklist—case reports.

Item	Moga 2024 [14]	Moga 2024 [15]	Muppa 2016 [52]	Maass 2018 [31]
1. Patient demographics are clearly described	Yes	Yes	Yes	Unclear
2. Patient history is clearly described	Yes	Yes	Yes	Unclear
3. Current clinical condition clearly described	Yes	Yes	Yes	Partial
4. Diagnostic tests/assessments clearly described	Yes	Yes	Yes	Unclear
5. Intervention clearly described	Partial	Yes	Partial	Unclear
6. Post-intervention clinical condition described	Yes	Yes	Partial	Partial
7. Adverse events identified/described	NA	NA	NA	NA
8. Key learning points are clearly provided	Yes	Yes	Yes	Partial
**Score (of 7 applicable items)**	**6/7—High**	**7/7—High**	**5/7—Moderate**	**2/7—Low**

**Table 3:** JBI Critical appraisal checklist—case series.

Item	Miller 2018 [13]	Bimstein 2018 [51]
1. Clear criteria for inclusion	Yes	Yes
2. Condition measured in a standard, reliable way	Yes	Yes
3. Valid methods used	Yes	Yes
4. Consecutive or clearly representative inclusion	Unclear	Unclear
5. Complete case series included	Unclear	Unclear
6. Clear reporting of demographics	Partial	Partial
7. Outcomes clearly reported	Partial (observational)	Partial (observational)
8. Statistical analysis appropriate	NA (descriptive)	NA (descriptive)
**Score**	**5/8—Moderate**	**5/8—Moderate**

**Table 4:** Confidence per AMSTAR-2 is low, thus studies’ conclusions about treatment efficacy are directionally informative.

AMSTAR-2 Domain for Guha Biswas 2023 [28]	Rating	Comment
PICO components pre-specified	Yes	PROSPERO registration CRD42019127469
Comprehensive literature search	Yes	Multiple databases; manual searching
Duplicate study selection	Partial yes	Single reviewer with second verification
Duplicate data extraction	No	Single extractor—**critical weakness**
List of excluded studies with justification	No	Excluded studies not individually listed—**critical weakness**
Adequate description of included studies	Yes	
Risk of bias in individual studies assessed	Partial yes	Limited quality appraisal reported
Adequate statistical methods (if meta-analysis)	NA	No pooling attempted
Risk of bias considered in interpretation	Partial yes	
Heterogeneity adequately investigated	NA	No pooling
Publication bias assessed	No	Not reported—**critical weakness**
Conflicts of interest declared	Yes	
**Overall AMSTAR-2 rating**	**CRITICALLY LOW confidence**	Three critical weaknesses were identified

**Table 5:** Evidence Table: Included studies on localized grade C molar-incisor pattern periodontitis in children aged 2–13 years (primary and mixed dentition, January 2014–April 2026).

Reference (PMID)	Study Design	Country	n (Primary/Mixed Dentition Subgroup)	Age Range (Dentition Phase)	Gender (M:F)	Treatment Strategy	Follow-Up	Drop-Outs	Key Outcomes: Disease Progression	Key Outcomes: Treatment Response	Notes/Limitations
Merchant et al., 2014 J Periodontol 85(12): 1722 PMID: 25167994	Prospective non-randomized cohort (NCT01330719); primary/mixed dentition subgroup reported separately	USA (Florida)	n = 22 (primary/mixed dentition from overall n = 97)	5–12 yrs, (primary and early mixed dentition)	Subgroup M:F proportional to overall 30M:66F; not separately reported	Full-mouth SRP + Amoxicillin 500 mg + Metronidazole 250 mg TID × 7 days; maintenance debridement at 3, 6, 12 months	12 months	Not reported for the primary/mixed dentition subgroup	Greater baseline bone destruction relative to age vs. permanent dentition group; rapid localized bone loss around primary first molars and deciduous incisors before treatment initiation	Significantly greater CAL gain in primary/mixed dentition group vs. permanent dentition (*p* < 0.05 at all timepoints); superior healing and regeneration potential in younger patients; significant PD reduction at 3, 6, 12 months	Strongest interventional evidence for primary/mixed dentition subgroup; permanent dentition subgroup (ages > 12 yrs) excluded from this table; single ethnic cohort (African American); no control group
Miller et al., 2018 J Clin Pediatr Dent 42(2):95 PMID: 29087795	Retrospective descriptive case series (characterization; observational only—no treatment intervention)	USA (Florida)	n = 33 (all primary dentition; prepubertal)	~3–9 yrs(primary dentition; mean age not stated)	Not reported	Observational/radiographic characterisation only—no treatment reported	Cross-sectional; no follow-up	N/A	Bone destruction concentrated around primary first molars and deciduous incisors; atypical external and internal root resorption; furcation involvement; premature tooth exfoliation; 90% of permanent LAP patients showed retrospective primary dentition bone loss	N/A—observational; no treatment outcomes	First systematic radiographic characterization of prepubertal LAP in primary dentition; confirms primary dentition disease precedes permanent dentition LAP; retrospective design; small n; no treatment data
Bimstein E., 2018 J Clin Pediatr Dent 42(2):91 PMID: 29087794	Retrospective radiographic case series (observational; companion paper to Miller et al. 2018 in same journal issue)	USA (Florida)	n = 29 children with aggressive periodontitis in primary/mixed teeth	Primary and mixed dentition (consistent with ages 4–12 yrs)	Not reported	Observational/radiographic characterisation only—no treatment intervention reported	Cross-sectional; no follow-up	N/A	Bone destruction predominantly at distal maxillary canines, proximal areas of first maxillary primary molars, and distal mandibular first primary molars; first primary molars are most frequently affected, followed by maxillary cuspids and second molars; distribution not significantly different by gender, ethnicity, or side	N/A—observational; no treatment outcomes	Companion radiographic characterization study to Miller et al. (2018); provides tooth-level distribution data for C-MIP in primary/mixed dentition; published in the same journal issue; limited by cross-sectional design and absence of treatment data
Kalash et al., 2015 Pediatr Dent 37(5):35 PMID: 26531074	Prospective interventional sub-study (NCT01330719; primary/mixed dentition-inclusive cohort)	USA (Florida)	n = 34 total LAP; primary/mixed dentition stratum ~5–12 yrs within broader 5–21 yr cohort; exact subgroup n not separately reported	5–21 yrs (primary/mixed dentition stratum: ~5–12 yrs)	Not specified	Full-mouth SRP + Amoxicillin 500 mg + Metronidazole 250 mg TID × 7 days	6 months	None reported	Significantly elevated systemic serum LPS at baseline vs. healthy controls; LPS hyper-responsiveness correlated with the number of affected sites and GCF RANKL; systemic endotoxemia reflects full-mouth periodontal pathogen burden	SRP + systemic antibiotics significantly reduced serum LPS at 6 months (*p* < 0.05); LPS reduction correlated with PD and CAL improvements; systemic antibiotic component critical for LPS suppression; reduction in systemic inflammatory burden	Key biological rationale study for systemic antibiotic adjunct; age-disaggregated LPS data not separately published for ≤12 yr stratum; no control group; direct link between subgingival dysbiosis, systemic endotoxemia, and RANKL-mediated bone destruction
Guha Biswas et al., 2023 Dent J (Basel) 11(7):171 PMID: 37504562	Systematic review (PRISMA 2020) PROSPERO: CRD42019127469 (searched to Dec 2022)	Multi-national (reviewing global literature)	3 interventional studies (n = 60); 12 case reports/series (n = 19); all primary/early mixed dentition periodontitis	~2–12 yrs (primary and early mixed dentition across included studies)	Mixed; not pooled	SRP ± systemic antibiotics (Amoxicillin, Tetracycline, Metronidazole); extraction of hopeless teeth; 1 study used local antibiotics	Variable: 4 weeks to 5 years across included studies	Not systematically reported; evidence-based heterogeneous	Untreated: irreversible attachment loss, premature tooth exfoliation, risk of spread to permanent dentition; rapidly progressive if untreated	SRP + systemic antibiotics produced PD reduction and CAL gain in all 3 interventional studies; early treatment linked to primary tooth preservation and protection of permanent dentition; no RCTs; evidence rated very low	First systematic review on treatment of periodontitis in primary teeth; only 3 non-controlled interventional studies (n = 60); heterogeneous diagnostic criteria; urgent need for RCTs
Moga et al., 2024 J Clin Med 13(1):266 PMID: 38202182	Case report (delayed diagnosis; disease progression documentation)	Romania	n = 1 (4-year-old Caucasian girl)	4 yrs (primary dentition)	Female	Topical Metronidazole gel only (during delayed-diagnosis phase); tooth lost before systemic treatment could be initiated	~6 months from first symptom to tooth exfoliation	N/A (single case)	Rapid progression: advanced bone loss around lower deciduous canine/7.3; tooth lost within 6 months of mobility onset; 4–6 months diagnostic delay; 4 clinicians misattributed presentation to hypophosphatasia, bacterial tests prelevated, blood tests prelevated, apical third external root resorption, good oral hygiene, A. actinomycetemcomitans and P. gingivalis present	Topical antibiotics are insufficient once advanced attachment loss is present; irreversible tooth/7.3 loss before systemic treatment; direct clinical cost of diagnostic delay documented	First documented Central/Eastern European case of prepubertal LPP; pathognomonic radiographic signs missed by 4 clinicians; underscores the need for an early periodontal diagnostic algorithm, the importance of early symptoms—early mild-to-moderate pains with no known origin
Moga et al., 2024 J Clin Med 13(16):4878 PMID: 39200016	Case report (1-year prospective follow-up of the same patient as in 2024)	Romania	n = 1 (4–5-year-old Caucasian girl)	4–5 yrs (primary dentition)	Female	Full-mouth SRP + Augmentin 1 g/day (500 mg BID) and Metronidazole 250 mg/day (125 mg DIB) × 10 days; topical antimicrobials; intensive oral hygiene reinforcement; 3-monthly monitoring	12 months post-treatment	N/A (single case; fully compliant)	One tooth/7.3 already lost before systemic antibiotic treatment; bacterial tests prelevated, elevated periodontopathic bacterial load, apical third external root resorption, good oral hygiene, A. actinomycetemcomitans and P. gingivalis present	Periapical bone regeneration radiographically evident at 2 months post-treatment (fastest regenerative response in any included study); gingival inflammation resolved; bacteria maintained at low levels; 8.3 with prior treatment, mobility regained physiological mobility, and with remaining primary teeth stable at 12 months	Radiologically documented bone regeneration within 8 weeks in a 4-year-old Caucasian patient in Central Europe; 10-day antibiotic course (vs. 7 days in Florida protocol); demonstrates osteogenic potential of immature alveolar bone, confirmed reservoir of bacteria present in the oral cavity during entire follow-up, with low scores of bacteria after systemic antibiotic treatment
Muppa R et al., 2016 Contemp Clin Dent 7(2):262 PMID: 27307682	Case report (single systemically healthy child; 18-month follow-up)	India	n = 1 (5-year-old male, primary dentition)	5 yrs (primary dentition; prepubertal)	Male	Full-mouth SRP + systemic antibiotics (Amoxicillin + Metronidazole; doses not stated for 15 days) + oral hygiene reinforcement; periodic professional mechanical teeth cleaning throughout follow-up	18 months	N/A (single case; compliant throughout)	Rapid attachment loss and bony defects around primary first molars and anterior teeth with little plaque; 5 mm pockets with furcal involvement; reported teeth loss, systemic workup normal; A. actinomycetemcomitans and P. gingivalis identified in tissue biopsy, blood test prelevated	Disease progression arrested at 18 months; bone loss controlled; no further exfoliation during follow-up; gingival inflammation resolved; successful outcome with combined antibiotic therapy and regular periodontal maintenance	First Indian case report of localized C-MIP in a systemically healthy child; expands ethnic/geographic evidence base; no quantitative PD/CAL data; 18-month follow-up is the longest among individual case reports in this table, when referred to a specialist, extremely high teeth mobility (52,53,62,63,54,55,64,65), high intensity pains, and gingival bleeding, reported bacterial reservoir present in oral cavity during the entire follow-up
Mass et al., 2018 Eur Arch Paediatr Dent 9(1):61–63 PMID: 29332208	Case report (single systemically healthy child; follow-up reported)	Israel	n = 1 (3-year-old male, primary dentition)	3 yrs (primary dentition; prepubertal	Male	Local application of 0.2% Chlorhexidine twice a day for 10 days. Scaling and curettage + with antibiotic therapy with Amoxicillin 250 mg TID × 7 days	26 months	N/A (single case; fully compliant)	Localized aggressive periodontitis/C-MIP in primary dentition; bone loss around primary teeth; 7.3 already lost and 8.3 with excessive mobility exfoliating within one month, disease progression documented pre-treatment, bacterial tests prelevated, blood tests prelevated, apical third external root resorption, good oral hygiene	Treatment response documented; under general anesthesia, a full-mouth scaling and curettage, clinical improvement reported following periodontal intervention, with stable conditions during the 26 months of follow-up	First documented Israeli case of prepubertal LPP; pathognomonic radiographic signs; both teeth/primary lower canines lost, multiple systemic antibiotic treatments history, no active bacterial reservoir present in oral cavity during the follow-up, no rebound reported, underscores the need for an early periodontal diagnostic algorithm. When referred to a specialist, no pain was reported

Abbreviations: SRP = Scaling and Root Planing; CAL = Clinical Attachment Level; PD = Probing Depth; LPS = Lipopolysaccharide; RANKL = Receptor Activator of Nuclear Factor κB Ligand; GCF = Gingival Crevicular Fluid; TID = Three Times Daily; RCT = Randomized Controlled Trial; N/A = Not Applicable; n = number of patients; M = Male; F = Female.

**Table 6:** Evidence Table: Quantitative and semi-quantitative clinical outcomes across included studies.

Study	n (≤13 yrs)	Follow-Up	Mean PD Reduction	Mean CAL Gain	Radiographic Outcome	Microbiological Outcome	Immunoinflammatory Outcome
Merchant et al., 2014 J Periodontol 85(12):1722	22	12 mo	Sig. at 3, 6, 12 mo (*p* < 0.05); mm not separately reported for primary dentition subgroup	Sig. greater than permanent dentition group (*p* < 0.05 all timepoints); mm not separately reported	Not specifically reported for the subgroup	Not reported	Not reported
Miller et al., 2018 J Clin Pediatr Dent 42(2):95	33	None (cross-sectional)	N/A	N/A	Vertical bone defects at primary first molars/incisors; atypical root resorption; furcation involvement	Not reported	N/A
Bimstein E., 2018 J Clin Pediatr Dent 42(2):91	29	None (cross-sectional)	N/A	N/A	Bone loss predominantly at the distal maxillary canine, proximal primary first molars, and distal mandibular first molar	Not reported	N/A
Moga et al., 2024 J Clin Med 13(1):266	1	~6 mo (to tooth loss)	N/A	N/A	Advanced bone loss around the lower deciduous canine	Not reported	N/A
Moga et al., 2024 J Clin Med 13(16):4878	1	12 mo	Resolution of gingival inflammation; no quantitative PD/CAL reported	Resolution of inflammation; no quantitative data	Periapical bone fill at 2 months; stable at 12 months	Low bacterial levels were maintained at 12 mo	N/A
Muppa R et al., 2016 Contemp Clin Dent 7(2):262	1	18 mo	Disease arrested; no quantitative data	No quantitative data	No further bone loss at 18 mo	Aa and P. gingivalis identified at baseline; not re-tested	N/A
Mass et al., 2018 Eur Arch Paediatr Dent 9(1):61–63 PMID: 29332208	1	26 mo	Not reported	Not reported	Not reported	Not reported	Not reported
Kalash et al., 2015 Pediatr Dent 37(5):35	~34 (subgroup n not separated)	6 mo	Not the primary outcome	Not the primary outcome	N/A	N/A	Serum LPS was significantly reduced (*p* < 0.05), correlated with PD/CAL improvement
Guha Biswas et al., 2023 Dent J (Basel) 11(7):171	60 (interventional) + 19 (case reports)	4 wks–5 yrs	PD reduction reported in all 3 interventional studies; pooled mean not calculable	CAL gain reported in all 3 interventional studies; pooled mean not calculable	Not systematically reported	Not systematically reported	N/A

## Data Availability

The original contributions presented in this study are included in the article. Further inquiries can be directed to the corresponding author(s).
